# Outcomes of Patients with Metastatic Melanoma—A Single-Institution Retrospective Analysis

**DOI:** 10.3390/cancers14071672

**Published:** 2022-03-25

**Authors:** Lidia Szatkowska, Jan Sieczek, Katarzyna Tekiela, Marcin Ziętek, Paulina Stachyra-Strawa, Paweł Cisek, Rafał Matkowski

**Affiliations:** 1 Clinical Department of Cardiology, 4th Military Hospital, Rudolfa Weigla 5, 50-981 Wrocław, Poland; 2 Department of Orthopedic Surgery, Provincial Specialist Hospital, Kamieńskiego 73A, 51-124 Wrocław, Poland; jjanskeczek@gmail.com; 3 Department of Oncology, Lower Silesian Oncology, Pulmonology and Hematology Center, Plac Hirszfelda 12, 53-413 Wrocław, Poland; katarzyna.tekiela24@gmail.com; 4 Department of Oncology, Wrocław Medical University, wyb. L. Pasteura 1, 50-367 Wrocław, Poland; wk-28@umed.wroc.pl (M.Z.); matkowski.rafal@dco.com.pl (R.M.); 5 Department of Surgical Oncology, Lower Silesian Oncology, Pulmonology and Hematology Center, Plac Hirszfelda 12, 53-413 Wrocław, Poland; 6 Department of Radiotherapy, Medical University of Lublin, Chodźki 7, 20-093 Lublin, Poland; paulina.stachyra@gmail.com (P.S.-S.); pcisek@interia.eu (P.C.)

**Keywords:** melanoma, treatment, prognosis, PD-1, PD-L1, CTLA4, BRAF inhibitors, MEK inhibitors, immunotherapy

## Abstract

**Simple Summary:**

Approximately 15% of patients diagnosed with locally advanced malignant melanoma will relapse. Currently, anti-PD-1 and anti-CTLA4 antibodies and BRAF/MEK inhibitors are the mainstay of treatment of advanced, inoperable or disseminated malignant melanoma. A group of 52 patients treated for disseminated malignant melanoma in 2013–2018 was subjected to a retrospective analysis. Overall survival, time to relapse after surgery, time to first disease progression after first-line treatment and time to second progression after second-line treatment were assessed. Factors such as age of the patients, type of progression, stage, blood parameters and the treatment used were analyzed for their influence on prognosis. The study showed that the BRAF V600 mutation is an independent prognostic factor for the recurrence of malignant melanoma after surgery. Patients with this mutation had a longer progression-free time compared to patients undergoing monotherapy with anti-PD-1/PD-L1 antibodies (*p* = 0.046). The type of treatment used had no effect on overall survival (Z = 0.23, *p* > 0.05).

**Abstract:**

Background: This study assessed risk factors and the results of treatment with anti-PD-1 antibodies and BRAF/MEK inhibitors for advanced malignant melanoma. Methods: A retrospective analysis was performed on 52 patients treated with immunotherapy and BRAF/MEK inhibitors for disseminated malignant melanoma. Results: The median follow-up was 31 months (6–108 months). The median PFS1 was 6 months (1–44 months). Second-line systemic treatment was applied in 27 patients (52%). The median PFS2 was 2 months (0–27 months), and the median OS was 31 months (6–108 months). Among the analyzed risk factors, only the presence of the BRAF mutation was statistically significant for disease recurrence after surgery. In patients undergoing anti-BRAF/MEK therapy, the median PFS1 was 7 months, and in patients undergoing mono-immunotherapy, 4 months. The 12- and 24-month PFS1 rates in the group treated with BRAF inhibitors were 29 and 7%, respectively, and in patients treated with mono-immunotherapy 13 and 0%, respectively (Z = 1.998, *p* = 0.04). The type of treatment used had no effect on OS (Z = 0.237, *p* > 0.05). Conclusion: Patients with the V600 mutation should be closely monitored. In the event of disease recurrence, treatment with BRAF/MEK inhibitors should be considered. The type of treatment used has no effect on OS.

## 1. Introduction

Malignant melanoma is a tumor that originates from melanocytic cells. It mainly affects the skin of the trunk and limbs but can also occur on mucous membranes and eye (uveal melanoma). In Poland, the incidence of melanoma is constantly increasing. An approximately threefold increase has been observed in the last three decades. The standardized incidence rates are 2.7/10^5^ for men and 3.9/10^5^ for women in the 20–44 age group. This coefficient increases with age. For patients aged 65+ the standardized incidence rates are 24.5/10^5^ and 39.7/10^5^ for men and women, respectively [1,2]. The median age at onset of malignant melanoma is 50 years, and the mortality rate is higher in men than in women (1.4 versus 1.2%) [3]. In the USA in 2017, 86,000 cases of melanoma were diagnosed and 8000 people died from the disease [4]. The annual increase in the incidence of melanoma in people with fair skin in the world amounts to 4–6% [5]. The most common risk factors are UV radiation of artificial and natural origin, chronic exposure to chemicals, as well as genetic factors, for example, a terminal mutation in CDKN2A (cyclin-dependent kinase inhibitor 2A). An advanced neoplastic process in the form of regional spread or generalized disease is diagnosed in approximately 15% and 5% of patients, respectively [6]. The basic and, at the same time, the most effective method of treating malignant melanoma is surgery. Adjuvant treatment benefits patients at increased risk of relapse. The use of such treatment reduces the risk of disease recurrence or death by 17–51% [6,7,8]. The first drug approved for adjuvant therapy in 1995 was recombinant interferon alfa-2b (Intron A). Interferon alfa (IFN-α) is a cytokine produced primarily by plasmacytoid dendritic cells as a result of stimulation of their TLR7 and TLR9 (Toll-like) receptors by cytokines. Binding of IFN-α to the receptor activated Janus-activated tyrosine kinase (JAK) results in an increase in immune system responses, inhibition of cell proliferation and stimulation of their differentiation [9]. The use of Intron A reduced the risk of recurrence by 17–18% (*p* <0.0001), and meta-analyses showed an improvement in 5-year survival by approximately 3–5% [8].The patients benefiting most from the use of interferon in adjuvant treatment are patients with an ulcerated primary lesion, with micro-metastases in the sentinel node, but without macro-metastases to clinically enlarged lymph nodes [10]. Before the era of monoclonal antibodies and molecularly targeted therapy, the mainstay of treatment of advanced inoperable or disseminated malignant melanoma was chemotherapy based mainly on dacarbazine, temozolomide or fotemustine and interleukin 2 immunotherapy [10]. An analysis of eight different studies showed that the average survival time for patients with stage IV melanoma treated with interleukin-2 was 11.4 months, and about 10% of patients survived 5 years [11]. In patients with complete lesion regression, the duration of response to treatment was even 40 months [11]. The median survival time in patients treated with dacarbazine in monotherapy is on average 6 to 7 months, and in the case of polychemotherapy with the BOLD regimen (bleomycin, vincristine, lomustine, dacarbazine, tamoxifen) or the CVD regimen (cisplatin, vincristine, dacarbazine), it averages between 7 and 12 months [12]. The addition of interferon and interleukin 2 to chemotherapy with the DBDT regimen (dacarbazine, carmustine, cisplatin, tamoxifen) increased the median OS from 9 to 11.3 months [13,14].

On neoplastic cells, as well as in the tumor microenvironment, there is an increased expression of molecules that inhibit the immune response of T lymphocytes, and a low expression of molecules causing an increase in this response. The immune response through T lymphocytes is complex and involves many steps and checkpoints. One such checkpoint is the programmed death-1 (PD-1) receptor and the CTL4 (cytotoxic T-lymphocyte antigen 4, CD152) inhibiting the body’s immune response. Blocking these molecules or their ligands results in an enhanced immune response and recognition of cancer cells as foreign [15,16]. Messenger proteins, overexpressed as a result of gene mutation or amplification, have also become molecular targets of new anticancer drugs. Interaction of messenger proteins with the drug interferes with the transmission of signals from molecules specific to the carcinogenesis process.

Research on the immune response in cancer and on cellular signaling pathways has resulted in the introduction of a new generation of drugs into treatment. Currently, anti-PD-1 (nivolumab, pembrolizumab) and anti-CTLA4 (ipilimumab) antibodies are commonly used in the treatment of malignant melanoma [9].

PD-1 is a surface protein expressed on T effector lymphocytes (cytotoxic CD8 and CD4 helper), B lymphocytes, NK cells and dendritic cells. Factors increasing the expression of PD-1 include cytokines (interleukins: IL 2, IL7, IL15, IL21) and estrogens [15]. The ligands for PD-1 are PD-L1 and PD-L2 molecules. Induction of the PD-L1 molecule occurs in most cells, while PD-L2 is associated primarily with dendritic cells, monocytes and non-hematopoietic tissues. Chronic antigenic stimulation leads to PD-1 overexpression on lymphocytes, which causes the effector lymphocytes to lose their cytotoxic capacity (exhausted lymphocytes), including the ability to kill tumor cells. Increased PD-1 expression is observed on T lymphocytes around the tumor and on cells belonging to the tumor microenvironment [16]. The binding of PD-L1 on tumor cells with the PD-1 receptor on lymphocytes or NK cells inhibits the immune response. Interactions resulting from the binding of tumor infiltrating lymphocytes (TIL) with the PD-L1 and PD-L2 ligands on tumor cells lead to impaired effector lymphocyte function. Overexpression of PD-L1 on neoplastic cells is most likely the result of both impaired signal transduction in intracellular pathways such as PI3k/Akt (phosphatidyl-inositol 3-kinases/serine/threoninekinase/protein kinase B), STAT3 (signal transducer and activator of transcription 3) or ALK (anaplastic lymphoma kinase) and the action of gamma interferon. Neoplastic cells secrete interleukin 10, which stimulates the expression of PD-L1 and reduces the expression of costimulatory molecules—CD80 on monocytes and CD86 on dendritic cells [15,16].

The CTLA4 molecule is present on active T lymphocytes and on regulatory T lymphocytes. The expression of CTLA4 on T cells depends on the CD28 receptor and the TCR/CD3 complex (T-cell receptor). TCR is the main pathway that stimulates T lymphocytes as a result of antigen presentation by major histocompatibility complex (MHC) cells [15]. The binding of CTLA4 to its ligands—CD80 and CD86 on dendritic cells—inhibits the activity of T helper cells and stimulates T-receptor cells, which results in the inhibition of the immune response. A competitor of CTLA4 for its ligands is the CD28 receptor, the stimulation of which leads to the stimulation of TCR, activation of the immune response and reduction of apoptosis by increasing the amount of the anti-apoptotic protein Bcl-xL. Increasing the proliferation of T lymphocytes stimulates the appearance of CTLA-4 by inhibiting CD28 feedback, reducing the proliferation of T lymphocytes (negative feedback). CD28 dysfunction has been described not only in malignant melanoma, but also in breast cancer, lung cancer and cervical cancer [17,18,19].

Another group of drugs used in the treatment of malignant melanoma are inhibitors of the RAS/RAF/MEK/ERK pathway. The Ras protein has GTPase activity and plays an important role in signal transduction to the MAP kinase cascade. Activating RAS also indirectly by binding PI3K (phosphatidylinositol 3 kinase) activates a different signaling pathway—PI3K/AKT/mTOR(phosphatidyl-inositol 3-kinases, serine/threoninekinase/protein kinase B/mammalian target of rapamycin kinase) [20]. The result of the stimulation of these signaling pathways is cell proliferation and differentiation. The first proteins activated by RAS are proteins belonging to the RAF family (serine/threonine-specific protein kinases). The RAF kinase family includes proteins such as ARAF, BRAF, and CRAF. Mutations in BRAF occur in approximately 50% of malignant melanomas, and 90% of them involve codon 600 and the substitution of valine with glutamic acid (V600E), and 5–6% the substitution of valine with lysine (V600 K). Other mutations in this codon are V600′E2′ (GTG > GAA) and BRAF V600D (GTG > GAT) [20,21]. The product of the BRAF gene is a protein with serotonin-tyrosine kinase properties. Activation of this protein results in the phosphorylation of MEK1 and MEK2, which activate ERK1 and ERK2, resulting in signal transduction to the cell nucleus and the induction of cell proliferation. BRAF activity is inhibited by negative feedback between MEK and ERK. A mutation in BRAF causes constitutive kinase activation and escape from negative feedback [20,21]. The conversion of valine to glutamic acid in codon 600 destabilizes hydrophobic interactions and, by shifting the highly conserved DFG motif (D-aspartic acid, F-phenylalanine, G-glycine), increases the basal activity of the BRAF protein. Such a mutant protein has a higher capacity to activate ERK kinases compared to the wild-type BRAF [22]. The consequence of this activation is uncontrolled proliferation, escape from apoptosis and concomitantactivation of MEK-dependent angiogenesis by stimulation of HIF-1α (hypoxia inducible factor 1-alpha) and VEGF (vascular endothelial growth factor). BRAF inhibitors (vemurafenib, dabrafenib, encorafenib) and MEK inhibitors (trametinib, binimetinib, cobimetinib) are used in molecularly targeted treatment of melanoma [9].

Their introduction to therapy has reduced the role that chemotherapy and non-specific active immunotherapy (interferon, interleukin 2) plays in the treatment of patients with advanced melanoma [9]. These drugs are used both in adjuvant therapy and in the treatment of inoperable melanoma [9]. The use of new-generation drugs in adjuvant treatment reduced the risk of disease recurrence from about 51% to 25% [7,23]. A phase III study investigated the efficacy of the combination of ipilimumab and dacarbazine in patients with unresectable malignant melanoma who had not received prior treatment. The OS for patients receiving both drugs was 11.2 months, compared to 9.1 months for the dacarbazine plus placebo arm [24].

Unfortunately, defining unambiguous criteria for the eligibility of patients for molecularly targeted therapy is extremely problematic, and, therefore, still constitutes a major challenge for modern medicine. Determining patient eligibility for modern treatment should be preceded by testing for the presence of changes in cellular metabolism or genetic profile, which may be a target for such treatment [9].

This study assessed risk factors and the results of treatment with anti-PD-1 antibodies, anti-CTLA4 antibodies and BRAF/MEK inhibitors for advanced malignant melanoma.

## 2. Materials and Methods

The study was conducted on a group of 52 patients treated with anti-PD-1 and anti-CTLA4 antibodies and BRAF/MEK inhibitors for disseminated malignant melanoma in 2013–2018 at the Lower Silesian Center of Oncology, Pulmonology and Hematology. The examined group included patients with disease progression in the form of local recurrence or distant relapses following a primary lesion surgery, as well as patients with disseminated primary neoplasms. Patients may have undergone surgery for local recurrence or distant metastases. Palliative radiotherapy was also allowed. Patient characteristics are presented in Table 1. All patients undergoing first-line immunotherapy were BRA-wildtype. Only 1 patient among these receiving anti-BRAF/MEK inhibitors was treated with a combination of two drugs. It was related to reimbursement regulations valid in Poland at the time. Overall survival (OS), time to relapse after surgery (RFS), time to first progression after first-line treatment (PFS1) due to disease relapse and time to second progression after second-line treatment (PFS2) were assessed. Factors such as age, type of progression, stage according to Clark classification, blood counts (HGB, MCV, PLT), blood biochemical values (LDH, AST, ALT) and applied treatment were analyzed statistically for their influence on prognosis.

The statistical analysis of selected factors was carried out using the Statistica software (version 13.1, StatSoft, Kraków, Poland). Comparisons between variables were performed through U-Mann–Whitney test. Comparisons between survival times were performed by the log-rank test. *p*-value < 0.05 was considered statistically significant.

## 3. Results

**Figure 1:**
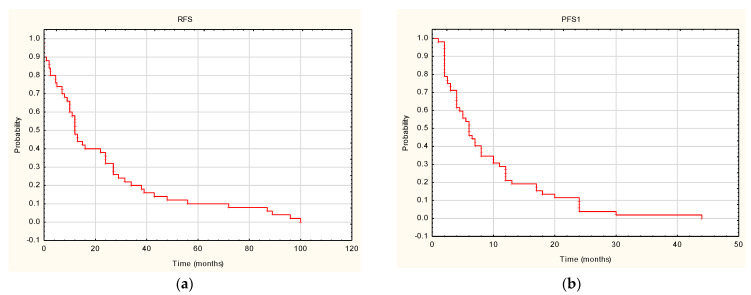

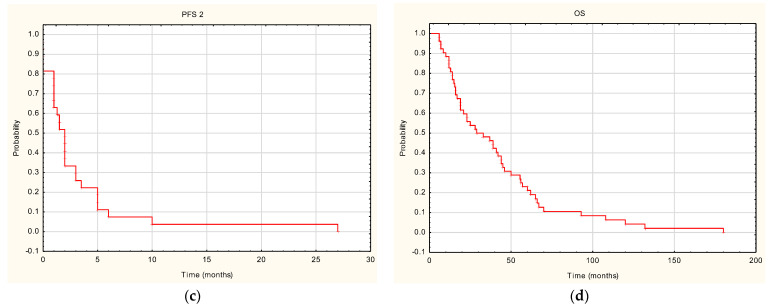
Survival outcomes: (**a**) time to relapse after surgery (RFS); (**b**) time to first progression after first-line treatment (PFS1); (**c**) time to second progression after second-line treatment (PFS2); (**d**) overall survival (OS).

The median follow-up time in the analyzed group of patients was 31 months (6–108 months). The median time to relapse (RFS) was 12 months (0–100 months) (Figure 1A). Progression occurred in the entire analyzed group. The median time to first progression was 6 months (1–44 months) (Figure 1B). Second-line systemic treatment was applied in 27 patients (52%). The median time to second progression (PFS2) was 2 months (0–27 months) (Figure 1C).Fifty-one patients (98%) from the analyzed group died during the follow-up. The median overall survival was 31 months (6–108 months), while the mean was 40.74 ± 35.31 months (Figure 1D).

**Figure 2:**
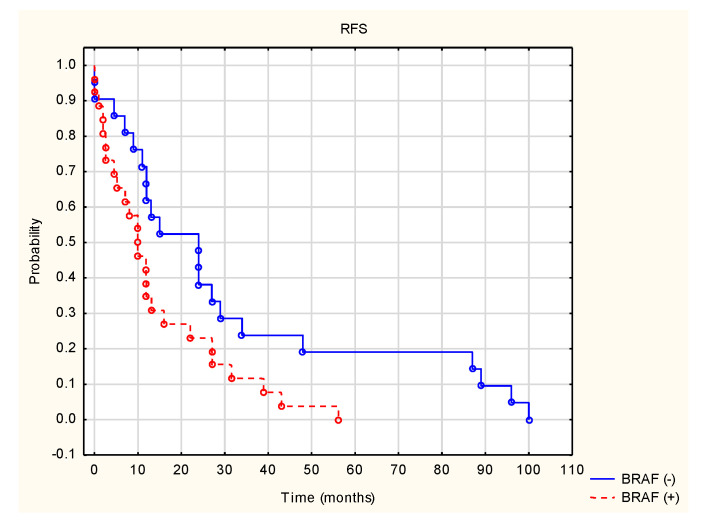
Time to relapse after surgery (RFS) in patients BRAF V600 (+) and wild-BRAF (−).

Among the analyzed risk factors, only the presence of the BRAF mutation was a statistically significant risk factor for disease recurrence after surgery (Figure 2). In patients with the BRAF mutation, the median relapse-free survival (RFS) was 10 months, while in patients without the BRAF mutation, it was 24 months. The 12- and 24-month RFS rates in the BRAF (+) group were 35% and 23%, respectively, and 62% and 38% in the BRAF (−) group, respectively. The result was statistically significant (Z = −2.21151, *p* = 0.027). The impact of other factors on survival parameters is presented in Table 2.

**Figure 3:**
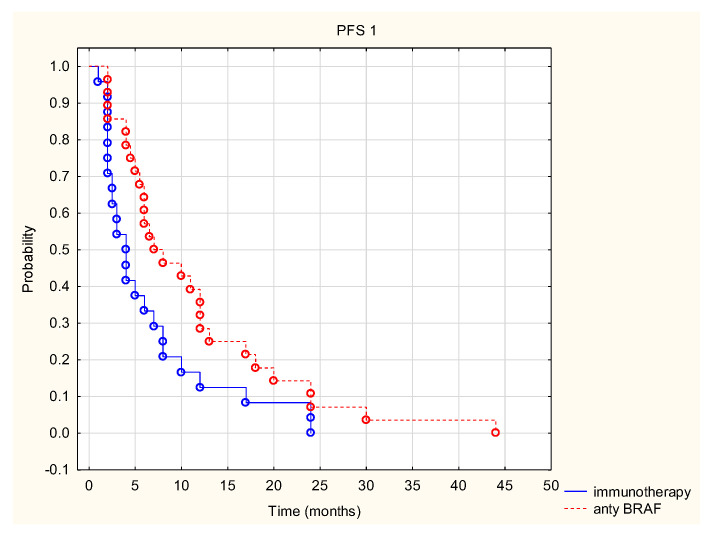
Time to first progression after first-line treatment (PFS1) in patients treated with anti-BRAF and immunotherapy.

The analysis shows the superiority of anti-BRAF therapy over mono-immunotherapy in terms of PFS1. In patients receiving targeted anti-BRAF therapy, the median PFS1 was 7 months, and in patients receiving mono-immunotherapy, 4 months. The 12- and 24-month PFS1 rates in the group treated with BRAF inhibitors were 29% and 7%, respectively, and in the group treated with immunotherapy, 13% and 0%, respectively. The results were statistically significant (Z = 1.998, *p* = 0.046) (Figure 3). The type of treatment used had no effect on overall survival (Z = 0.237, *p* > 0.05).

## 4. Discussion

Inter- and intracellular communication underpins the regulation of mechanisms responsible for fundamental life functions, such as cell growth, division, differentiation and death [25]. The pathological increase or decrease in the activity of some molecules involved in these processes underlies tumor progression. The concept of targeted therapy is a significant progress in the individualization of anti-cancer treatment because, according to its premise, the choice of an appropriate therapeutic strategy depends on the genetic predisposition of the patient, which is determined by the presence or absence of a specific molecular target characteristic for a given drug [26]. There are also additional factors related to gender, age, race or tumor histology, the overall assessment of which should constitute the basis for determining treatment eligibility.

Ipilimumab is an IgG1k monoclonal antibody that blocks CTLA4, which increases access of CD80/86 ligands to the CD28 receptor, resulting in an increase in both proliferation of T cells and immune response. The lack of CTLA4 inhibition of the CD28 receptor also increases the amount of the anti-apoptotic protein Bcl-xL. Ipilimumab was the first drug for which prolonged survival and remission times have been shown in metastatic malignant melanoma [9]. In 2011, ipilimumab was registered for the treatment of inoperable or disseminated malignant melanoma in previously treated patients, based on the results of the MDX010-20 study [18,27]. The median OS was 10 months in the ipilimumab arm with the gp100 vaccine (95% CI, 8.5–11.5), 10.1 months with ipilimumab monotherapy (95% CI95, 8.0–13.8), and 6.4 months (95% CI 5.5–8.7) in the group of patients receiving only gp100. Additionally, the objective response rate was highest in the ipilimumab monotherapy arm at 10.9% (95% CI; 6.3–17.4) vs. 5.7% (3.7–8.4) for gp100 plus ipilimumab and 1.5% (0.2–5.2) for gp100 [18]. In 2015, ipilimumab was registered for adjuvant treatment as well, based on a study in which the ipilimumab group achieved an average remission of 26 months compared with 17 months in the placebo group. It was also associated with an increase in survival [7].

Nivolumab and pembrolizumab are human IgG4 monoclonal antibodies directed against the PD-1 receptor that block its interaction with PD-L1 and PD-L2 ligands. They are used in the treatment of malignant melanoma, but also in kidney, liver, head, neck and lung cancer [9]. A randomized phase III trial compared the efficacy of nivolumab to ipilimumab in the adjuvant treatment of patients with stage IIIB, IIIC and IV malignant melanoma. A longer progression-free survival and less frequent serious side effects have been demonstrated in the case of nivolumab. The 12-month relapse-free rate was 70.5% for nivolumab and 60.8% for ipilimumab [27]. This study became the basis for the FDA approval of nivolumab in 2017 for the adjuvant treatment of patients with advanced melanoma [28]. In the CheckMate-067 trial, the combination of nivolumab and ipilimumab in first-line treatment of advanced melanoma was shown to prolong PFS and OS. The mean PFS in the nivolumab and ipilimumab group was 11.5 months and was significantly longer than in the nivolumab or ipilimumab monotherapy groups (6.9 months and 2.9 months, respectively). In the 6.5-years follow-up, the mean OS in the group receiving nivolumab and ipilimumab was 72.1 months, while in the groups receiving either nivolumab or ipilimumab monotherapy, it was 36.9 months and 19.9 months, respectively [29].

In our study, most patients in first-line treatment received ipilimumab (10 patients) or pembolizumab (10 patients). Only four patients were treated with nivolumab. The median PFS1 was 7 months and was similar to that of the nivolumab group in the CheckMate-067 trial. The 12- and 24-month PFS1 rates were 13% and 0%, respectively. All patients were BRAF-wildtype.

Pembrolizumab is registered for both the treatment of advanced disseminated malignant melanoma and the adjuvant treatment of patients with lymph node involvement [9]. In the KEYNOTE-006 trial, in advanced unresectable malignant melanoma in previously treated or untreated patients, the median OS was 32.7 months in the pembrolizumab arm and 15.9 months in the ipilimumab arm. The 5-year OS rate (95% CI) was 38.7% (34.2–43.1) and 31.0% (25.3–36.9), respectively. For patients receiving first-line treatment, the median OS was 38.7 months in the pembrolizumab arm, compared with 17.1 months in the ipilimumab arm (*p* = 0.0036). In patients receiving second-line treatment, the median OS was 23.5 vs. 13.6 months (*p* = 0.36), respectively [28]. Another study, KEYNOTE-001, which also investigated the use of pembrolizumab in previously treated and untreated malignant melanoma patients, showed an ORR of 33%, a 12-month PFS of 35%, and a median OS of 23 months. Forty-four percentof patients responded for more than a year. The ORR for patients who progressed within 24 weeks of their last ipilimumab dose was 24%. Objective responses were also obtained in patients regardless of the presence of the BRAF V600 mutation and irrespective of prior treatment with BRAF inhibitors [30].

RAS/RAF/MEK/ERK pathway inhibitors include trametinib, vemurafenib, dabrafenib, and cobimetinib, among others. All of these drugs are approved for the treatment of unresectable or metastatic malignant melanoma with the BRAF V600 mutation. Vemurafenib and dabrafenib provide potent selective inhibition of kinase activity in BRAF V600-mutant melanoma by blocking ERK phosphorylation and cellular proliferation. Vemurafenib and dabrafenib are only effective in BRAF V600-mutant melanoma. In contrast, encorafenib also displays some inhibitory effect in wild type BRAF [31]. Cobimetinib and trametinib are highly selective MEK 1 and 2 inhibitors that block signal transduction to the ERK [32].

A phase III trial (BREAK3) compared dabrafenib with dacarbazine in BRAF V600 E patients with unresectable or metastatic melanoma. The median PFS was 5.1 months in the dabrafenib arm, compared to 2.7 months in the dacarbazine arm, and the ORR was 52% to 17%, respectively [33]. A similar study was conducted with vemurafenib. It showed that the use of vemurafenib resulted in a 74% reduction in the risk of progression compared to dacarbazine [34].

Combination therapy with dabrafenib and trametinib leads to inhibition of two kinases in the RAS/RAF/MEK/ERK pathway (constitutively activated by oncogenic mutations in BRAF). The COMBI-d and COMBI-v trials compared the efficacy of treatment with two MEK and BRAF inhibitors (dabrafenib and trametinib) to monotherapy with dabrafenib or vemurafenib in patients with unresectable cutaneous melanoma (stage IIIC) or metastatic cutaneous melanoma (stage IV) with the BRAF V600 E/K mutation. Both studies demonstrated the superiority of the combination therapy over monotherapy. Thirty-four percent of patients in the dabrafenib plus trametinib group survived for 5 years, compared to 27% in the dabrafenib group and 23% in the vemurafenib group. Five-year PFS was also higher in the dabrafenib and trametinib arm (19% vs. 13% and 9%, respectively) [35]. A phase III trial showed that concurrent use of cobimetinib in combination with vemurafenib in patients with BRAF V600-mutant metastatic malignant melanoma results in prolongation of PFS, compared to vemurafenib monotherapy (9.9 months and 6.2 months, respectively) [36]. The addition of atezolizumab, an anti-PD-L1 monoclonal antibody, to the signaling pathway inhibitors (cobimetinib and vemurafenib) resulted in a prolonged time to progression (median PFS = 15.1 months in the atezolizumab arm vs. 10.6 months without atezolizumab, HR = 0.78; CI: 0.63–0.97; *p* = 0.025) [37].

Factors influencing prognosis in patients with cutaneous melanoma without distant metastases include the thickness of the infiltrate, the presence of ulceration of the primary lesion, the presence of the N feature and the level of LDH [2,6]. In the case of regional lymph node metastases, prognostically significant factors include the number of lymph nodes involved, the type of metastasis—micro-metastases or macro-metastases—infiltration beyond the lymph node capsule and microsatellite as a component of the N feature. The presence of regional lymph node metastases is expected to be the most important determinant of prognosis in patients with cutaneous melanomas [2,6].

In the conducted analysis, the median OS was 31 months, while the mean was 40.74 ± 35.31 months. Median time to relapse (RFS) was 11.5 months (0–96 months). Median time to first progression (PFS1) was 6 months (1–44 months), and median time to second progression (PFS2) was 2 months (0–27 months). The only statistically significant risk factor for disease recurrence after surgery was the BRAF mutation. When the BRAF V600 K mutation was present, the median relapse-free survival (RFS) was 10 months, compared to 24 months in patients without the BRAF mutation. The 12- and 24-month RFS rates were also lower in the BRAF (+) group, compared to BRAF (−) (35% and 23% vs. 62% and 38%, respectively (Z = −2.21151, *p* = 0.027)).

The influence of BRAF mutation on the survival of patients with melanoma is not clearly defined. Many studies show a worse prognosis in patients with mutations in the RAF family genes [38,39]. The absence of BRAF or NRAS mutations is expected to be a better prognosis factor in stage III patients (20.0, 1.0–1000.0: 16.7, 0.6–1000.0, respectively) [39]. Patients with BRAF mutations are more likely to have regional lymph node metastases and ulceration in the primary lesion [22,40]. An analysis of 437 patients in stages I and II showed a positive correlation between the presence of the BRAF V600 E mutation and the young age of patients, and sometimes with the onset of metastases, with no effect on overall survival (*p* = 0.119). Of the patients who developed distant metastases, 51.7% had the mutation, compared to 36.7% of patients without disease recurrence (*p* = 0.031). Despite the lack of influence on overall survival, a trend was observed for shorter survival of patients without distant metastases (*p* = 0.061) and with BRAF mutation, compared to patients with the wild-type BRAF gene [41].

The KEYNOTE-006 trial demonstrated a shorter OS in patients with the BRAF V600 mutation in the tumor, compared to patients without this mutation (median OS 13.9 vs. 28.1 months, HR = 0.73, *p* = 0.0048) [7]. In a meta-analysis of four studies (674 patients), Safaee Ardekani et al. [42] found a higher risk of death from melanoma in the presence of the V600 E mutation in patients with stage IIIB and IIIC (HR = 2.25; 95% CI, 1.82–2.83). Contrary to the results presented in the paper cited above, a study conducted on the Polish population did not show any association between the BRAF mutation and time to progression [43]. Long et al. [44] demonstrated an association between the occurrence of BRAF mutations and the patient’s age at the time of primary tumor diagnosis (≤50 years). Distant metastases also occurred later in life in wild-type patients than in patients with BRAF mutation (56 years vs. 63 years). The time between the diagnosis of malignant melanoma and the occurrence of distant metastases did not differ between the two patient groups, but the median survival of patients with newly diagnosed metastatic melanoma was 5.7 months in patients with BRAF mutation (patients not treated with BRAF inhibitors), compared to 8.5 months in patients without the mutation. The median was not reached in patients treated with BRAF inhibitors [44]. The analysis of the group of patients shows that in patients undergoing targeted anti-BRAF therapy, the median PFS1 was 7 months, and in patients undergoing mono-immunotherapy the median PFS1 was 4 months (Z = 1.998, *p* = 0.046). Patients undergoing first-line immunotherapy were BRAF-wildtype. It should be noted that in this case patients with BRAF V600 mutation had a longer time to progression in comparison to BRAF-wildtype. That may indicate that the occurrence of this mutation can be associated with a better response to treatment. However, the type of treatment did not affect overall survival (Z = 0.237, *p* > 0.05). The discrepancy with the results of the DREAMseq study (EA6134) may be due to the fact that patients in the analyzed group were treated with anti-PD-1 or anti-CTLA4 mono-immunotherapy. In the DREAMseq trial, patients received a combination of anti-PD1 and anti-CTLA4 [45]. Therefore, many factors must be taken into account when establishing treatment eligibility of patients with the BRAF V600 mutation.

## 5. Conclusions

The BRAF V600 mutation is an independent prognostic factor for the occurrence of relapse after surgery. Patients with this mutation should be closely monitored. In the event of relapse, treatment with BRAF/MEK inhibitors should be considered when determining eligibility for treatment. The type of treatment used had no effect on overall survival (Z = 0.237, *p* > 0.05).

## Figures and Tables

**Table 1:** Patient characteristics.

Patient Parameters	Patient Data
Number of patients	52
**Sex**	
Women	15
Men	37
Median age (range)	59.9 (29–65)
**Type of recurrence**	
Distant	33
Local	2
Distant and local	16
**Location of primary lesion**	
Peripheral (extremities)	25
Central (head and torso)	8
Unknown primary	19
**BRAF status**	
BRAF V600	28
BRAF-wildtype	21
Unknown	3
**Blood parameters**	
HGB ≥12.5 g/dL	35
HGB <12.5 g/dL	17
MCV 80–95 fl	39
MCV >95 fl	5
MCV <80 fl	8
MCH 27–32 pg	41
MCH >32 pg	3
MCH <27 pg	8
MCHC 27–32 g/dL	48
MCHC >32 g/dL	3
MCHC <27 g/dL	0
PLT 150.000–450.000	48
PLT >450.000	4
PLT <150.000	0
LDH ≤240 U/L	27
LDH 241–480 U/L	16
LDH >481 U/L	3
Unknown LDH	6
ALT ≤40 U/L	49
ALT 41–200 U/L	3
AST ≤40 U/L	48
AST 41–200 U/L	4
**Level of invasion (Clark classification)**	
I	1
II	1
III	4
IV	24
V	14
**Metastases**	
Lymph nodes	38
Lung	30
Brain	18
Skin and subcutaneous tissue	18
Liver	16
Bones	10
Other	15
**Primary surgical treatment**	47
**First-line treatment**	52
**Anti-BRAF/MEK**	28
Vemurafenib	24
Dabrafenib	3
Vemurafenib + cobimetinib	1
**Anti-PD-1/anti-CTLA4**	24
Pembrolizumab	10
Ipilimumab	10
Nivolumab	4
**Second-line treatment**	27
Ipilimumab	16
Nivolumab	6
Pembrolizumab	5
**Other treatment**	
Palliative radiotherapy	23
Surgical treatment of metastases	14
Surgical treatment of local recurrence	11

Abbreviations: HGB—Hemoglobin, MCV—Mean corpuscular volume, MCH—Mean corpuscular hemoglobin, MCHC—Mean cell hemoglobin concentration, PLT—Platelet, LDH—Lactate dehydrogenase, ALT—Alanine aminotransferase, AST—Aspartate aminotransferase.

**Table 2:** The influence of variables on survival parameters.

Analyzed Parameter	RFS		PFS 1		PFS 2		OS	
	Test Value	*p*	Test Value	*p*	Test Value	*p*	Test Value	*p*
Sex	1.058	0.289	1.815	0.069	1.726	0.084	0.720	0.471
Age	0.076	0.782	0.187	0.664	0.506	0.476	0.708	0.400
Type of recurrence	0.837	0.402	−0.409	0.682	0.055	0.955	1.740	0.081
Location of primary lesion	−0.208	0.835	0.274	0.7831	−0.382	0.701	0.134	0.892
*BRAF* status	−2.211	0.027	1.266	0.205	0.546	0.584	−0.530	0.595
HGB (<12.5 vs. ≥12.5 g/dL)	−0.265	0.790	−1.312	0.189	−1.553	0.120	−1.415	0.157
MCV (norm vs. beyond the norm)	1.224	0.542	0.624	0.731	1.280	0.527	0.534	0.765
MCH (norm vs. beyond the norm)	0.245	0.884	1.566	0.457	5.065	0.079	0.587	0.745
MCHC (norm vs. beyond the norm)	−0.456	0.648	−1.132	0.257	−0.701	0.482	−0.804	0.421
PLT (norm vs. beyond the norm)	0.771	0.440	−0.833	0.404	−0.354	0.722	−0.283	0.776
LDH (continuous value)	0.128	0.719	1.582	0.208	2.051	0.152	1.946	0.162
Primary surgical treatment	−1.630	0.103	0.182	0.856	−0.741	0.458	−0.748	0.454
Level of invasion (Clark classification)	4.977	0.418	2.447	0.784	NA	NA	3.322	0.650
Type of treatment (anti-BRAF vs. immunotherapy)	NA *	NA	1.998	0.046	NA	NA	0.238	0.812
Lung metastases	NA	NA	NA	NA	NA	NA	0.521	0.602
Liver metastases	NA	NA	NA	NA	NA	NA	0.176	0.860
Brain metastases	NA	NA	NA	NA	NA	NA	0.344	0.730
Lymph node metastases	NA	NA	NA	NA	NA	NA	−0.389	0.697
Skin and subcutaneous tissue metastases	NA	NA	NA	NA	NA	NA	−0.003	0.997
Bone metastases	NA	NA	NA	NA	NA	NA	0.224	0.823
Other	NA	NA	NA	NA	NA	NA	−0.024	0.980

* NA (not applicable)—Thetest cannot be performed due to the small number of complete observations.
